# Longitudinal Study of Maternal Beliefs About Infant Crying During the Postpartum Period: Interplay With Infant’s Temperament

**DOI:** 10.3389/fpsyg.2021.786391

**Published:** 2021-12-16

**Authors:** Daiki Hiraoka, Michio Nomura, Masaharu Kato

**Affiliations:** ^1^Research Center for Child Mental Development, University of Fukui, Fukui, Japan; ^2^Japan Society for the Promotion of Science, Tokyo, Japan; ^3^Graduate School of Education, Kyoto University, Kyoto, Japan; ^4^Center for Baby Science, Doshisha University, Kyoto, Japan

**Keywords:** infancy, maternal beliefs, parenting, infant crying, longitudinal study, temperament

## Abstract

Infant crying is an important signal for their survival and development, and maternal beliefs about crying predict responsiveness to crying. Most studies have considered caregivers’ reactions to crying to be fixed, and it is unclear how they change with their caregiving experience. Additionally, it has recently been suggested that there is a bidirectional relationship between changes in mothers’ beliefs about crying and infants’ temperament. This study examined that relationship using a longitudinal study design. Maternal beliefs about crying and infant temperament of 339 Asian first-time mothers (mean age = 28.7 years, SD = 4.1) were measured at 1-month intervals over 4 months. There were 289 participants in Wave 2, 240 in Wave 3, and 164 in Wave 4. Prior to the main survey, we conducted a pre-survey to confirm the reliability and validity of the Japanese version of the Infant Crying Questionnaire. The results showed that parent-oriented beliefs, which focus on the caregiver rather than the crying infant, increased in mothers who had infants aged 3 months or older at Wave 1. We also found that the process of change in maternal beliefs was not uniform, and that infants high on surgency predicted changes in maternal beliefs about infant crying. Longitudinal studies of caregivers’ changes, such as the present study, are expected to contribute to understanding the co-development of caregivers and infants.

## Introduction

### Infant Crying

Infant crying is the primary means of communicating feelings and needs to the parent (Soltis, 2004). Mothers are specifically drawn to their infant’s cues (Thompson-Booth et al., 2014a), which motivates caring behavior and leads to infant survival. Furthermore, the maternal sensitivity to crying is also involved in the formation of infant-mother attachment security. Research has found that sensitive parenting behavior toward distressed infants predicted the development of infant secure attachment (Bell and Ainsworth, 1972; McElwain and Booth-Laforce, 2006). In early studies of attachment, the frequency of crying increased as the number of ignored crying episodes increased (Bell and Ainsworth, 1972). Attachment theorists emphasize providing prompt responses to infant crying according to infants’ needs and develop interventions to increase maternal sensitivity to infant crying (e.g., Hepworth et al., 2020).

### Changes in Maternal Responsiveness to Infant Crying

It is thought that changes in hormones, brain structure, and brain function occur through pregnancy and childbirth (e.g., Barrett and Fleming, 2011). These changes are a preparatory stage for responding sensitively to infant crying. Some studies have sought to evaluate the differences between mothers’ and non-mothers’ responses to infant crying (Sagi, 1981; Seifritz et al., 2003). Functional brain imaging studies showed that mothers while listening to infant crying, had greater responses in the auditory cortex, amygdala, and supplementary motor cortex, which are involved in discriminating the emotional valence of auditory stimuli and parenting behaviors (Seifritz et al., 2003; Parsons et al., 2017; Witteman et al., 2019). Furthermore, animal studies have also shown that non-mother mice engaged in avoidance behaviors to an infant distress call, but mother mice approached (Ehret et al., 1987). The physiological changes of mothers facilitate sensitivity to infant cues and seem to develop a maternal approach behavior that benefits the child’s survival.

There is, however, limited research on the changes in mothers’ responses to crying after becoming mothers. A few months postpartum, it has been suggested that a mother should not always react sensitively to infant crying, based on qualitative studies of interviews with mothers (Drummond et al., 1993; Kurth et al., 2014). It has been reported that first-time mothers try to find reasons for crying based on superficial information such as the infant’s facial expression and muscle tension, and try to stop crying by trial and error (Drummond et al., 1993; Kurth et al., 2014). Mothers gradually become able to discriminate the urgency and reason behind the crying implicitly, and the trial-and-error reactive response disappears in the first few months after delivery. Kurth et al. (2014) also found that postpartum mothers can manage their impulsive and obligatory feelings to stop their infant’s crying. The authors further suggested that with accumulated experience, mothers develop a self-soothing attitude and learn to relieve their stress in response to infant crying. The authors indicated that the accumulation of parenting experiences created a mental list of reasons for infant crying and ways to cope with crying, which may help reduce obligatory feelings and stress as a parent. Specifically, it was suggested that although mothers sought to practice perfect parenting and immediately tried to stop their infants from crying in the beginning, gradually they come to understand that infants have unstable periods and sometimes cry for no reason, and that it is not dangerous to leave them crying for a little while. In addition, it was pointed out that by decreasing the excessive concern for parenting advice from others, mothers would be able to establish their own parenting style and be more relaxed in dealing with crying. These observations seem reasonable considering that the cause of infant crying also changes from an urgent signal for survival to a signal for social communication (Nakayama, 2010). Similar results have been obtained in a recent quantitative study. Based on a sample of 178 mothers, 63% reported they would not leave their infant to “cry it out” at term, but the percentage dropped to 38% at 3 months postpartum (Bilgin and Wolke, 2020). However, no studies have statistically modeled the changes in maternal attitudes or responses to infant crying. Furthermore, the process of change may not be uniform. During pregnancy and the postpartum period, mothers experience various psychological and neurological changes; however, there are individual differences in the process of these changes (Kim et al., 2010; Planalp et al., 2013; Hoekzema et al., 2017). Though various psychological and social factors are involved in the responses of mothers to infant crying (Zeifman and St James-Roberts, 2017), it is unclear whether such individual differences exist immediately after childbirth, become apparent with postpartum experience, or both. In this study, we examined the alteration process of the response to crying and individual differences in this process through longitudinal data modeling.

### Infant Temperament and Parenting

We focused on infant temperament as a factor explaining the individual differences in mothers’ change pattern in response to infant crying. Parenting behavior is formed by the caregiver’s characteristics and the child’s temperament (Belsky, 1984; Taraban and Shaw, 2018). In this context, parenting and infant temperament influence each other bidirectionally rather than unidirectionally (Taraban and Shaw, 2018). It was thought that it would be helpful to incorporate simultaneous changes in infant development and the bidirectional relationship between maternal attitudes to infant crying and infant temperament into the model. In infancy, temperament is an individual difference in the child’s reactivity and self-regulation to a given environment (Rothbart, 2012).

In parenting research, three broad dimensions of infant temperament have been especially well-studied: surgency, negative emotionality, and orienting (Taraban and Shaw, 2018). Surgency, which describes differences in the general energy level and frequency of movement, required more challenging care of infants when surgency is high (McBride et al., 2002). In a previous study, higher infant surgency was associated with a subsequent decrease in caregiving by mothers (Planalp et al., 2013). Negative emotionality captures a tendency to experience sadness, frustration, fear, and anger. Studies indicate that mothers find it challenging to manage infant negative emotionality (Gauvain and Fagot, 1995; Crockenberg and Leerkes, 2003). Scaramella et al. (2008) examined the causal relationship between infant temperament and parenting behaviors at 12 and 24 months postpartum. The results indicated that mothers’ harsh parenting at 12 months of age predicted increases in children’s observed negative emotionality from 12 to 24 months. Children’s negative emotionality also predicted a decline in mothers’ supportive parenting behavior from 12 to 24 months. The third factor, orienting, refers to individual differences in self-regulation and the control of reactivity. In a recent longitudinal research study, mothers’ higher authoritative parenting style predicted later infants’ greater orienting, while higher maternal permissive parenting style predicted later infants’ lower orienting. Conversely, infant orienting was shown to positively predict the mother’s subsequent permissive parenting style (Wittig and Rodriguez, 2019). Bridgett et al. (2009) also showed that initial lower levels of infant orienting predicted more negative parenting 14 months later. Although each type of infant temperament has been shown to influence parenting style and attitudes and to develop along with them, few studies have examined the longitudinal relationship between the changes in mothers’ attitudes to infant crying and infant temperament.

### The Current Study

The primary purpose of this study was to statistically model the change in attitudes toward infant crying with parenting experience. We used the Infant Crying Questionnaire (ICQ; Haltigan et al., 2012), a questionnaire that measures adult’s beliefs and response intention to infant crying, which divides beliefs into infant-oriented and parent-oriented beliefs. Infant-oriented beliefs are beliefs that care about the crying infant and focus on the infant, while parent-oriented beliefs focus on the caregiver. Through interviews with mothers, Drummond et al. (1993) found that mothers shifted from an infant-centered response to a parent-centered response by 16 weeks postpartum when they heard crying. Given that both beliefs have a unique influence on the sensitive response to infant crying (Leerkes et al., 2015), it would be helpful to assess attitudes toward crying from these two aspects by using the ICQ. First, we examined the cross-sectional association between monthly infant age and each ICQ score using regression analyses. Next, to examine how attitudes toward crying change within individuals, the ICQ was administered at four different times, and the scores were modeled using the latent growth model (LGM). In this analysis, the latent variables of the intercept and slope are constructed to predict the repeatedly observed variables. If the variance of the latent slope variable is significant, it means that there is an individual difference in the process of change. In this regard, the current study employed two methods to elucidate individual differences: multivariate parallel growth modeling (parallel LGM) and growth mixture modeling (GMM). The parallel LGM models two or more LGM simultaneously and examines the covariate relationships among their latent variables. In this study, a questionnaire on infant temperament (the short form of the Japanese version of the Infant Behavior Questionnaire-Revised (IBQ-R; Nakagawa et al., 2009) was also administered as well as the ICQ, and the longitudinal bidirectional relationship between attitudes toward crying and infant temperament was examined. In the GMM, while the basic LGM assumes only one cluster, it is possible to assume latent clusters and examine whether the intercept and slope can be divided into two or more groups. Therefore, we examined the following hypotheses.

Hypothesis 1: Maternal beliefs about infant crying would change within individuals. In particular, infant-oriented beliefs would decrease, and parent-oriented beliefs would increase as the child develops. In addition, there would be individual differences in mothers’ initial beliefs and the degree of change in their beliefs.

Hypothesis 2: Infant’s temperament would be related bidirectionally to changes in maternal beliefs about crying. In particular, high surgency, negative emotionality, and low orienting would be associated with decreased infant-oriented beliefs and increased parent-oriented beliefs.

## Materials and Methods

### Ethics Statement

The study protocol was approved by the Ethics Committees of Graduate School of Education, Kyoto University. All participants provided informed consent for participation in this study.

### Participants

The questionnaire survey was conducted four times (four waves). The interval between each wave was approximately 1 month (September to December 2019). All data were collected from a web-based nationwide survey through Macromill, a marketing research company with monitors throughout Japan. The initial sample consisted of 339 Japanese-speaking mothers. There were 289 participants in Wave 2, 240 in Wave 3, and 164 in Wave 4. All participants were first-time mothers and their children were less than 4 months of age at Wave 1. To determine the sample size, we consulted the simulation, in which the effect of sample size on the result of LGM was calculated (Hamilton et al., 2003). The simulation results showed the effect of sample size on various parameters of LGM, such as convergence rate and root mean square error of approximation (RMSEA) on each number at the measurement point (4, 5, and 6 time points). The results showed that for the four time points LGM, the RMSEA was below 0.05 and the convergence rate was above 95% if the sample size was above 100 under all conditions. Allowing for attrition, we sampled over 300 participants at Wave 1, which resulted in a sufficient sample size by Wave 4. Participants who chose the same option for all questions were excluded from the analyses; two participants were excluded from Wave 1, none from Wave 2, one from Wave 3, and one from Wave 4.

### Procedure

The marketing research company selected first-time mothers with 0-4 months old infants, from its pool of monitors. No other exclusion criteria were established. The questionnaires were distributed to mothers who agreed to participate in the 4-wave longitudinal survey. Responses were solicited for a period of 1 week. The questionnaires were distributed again to the mothers who participated in the previous survey every other month; in total, 164 mothers responded at all four time points. The demographic composition of the participants and their infants is presented in Table 1. The participants’ sociodemographic characteristics did not differ across waves.

**TABLE 1:** The sociodemographic composition of the samples.

Characteristic	Wave1, *N* = 339	Wave2, *N* = 289	Wave3, *N* = 240	Wave4, *N* = 164	*p*-value^1^
Mother’s age, mean (SD)	28.7 (4.1)	28.9 (4.1)	29.0 (4.1)	29.1 (4.1)	0.79
Child’s age, mean (SD), minimum-maximum	1.83 (1.40), 0.00–4.00	2.92 (1.41), 1.00–5.00	3.99 (1.42), 2.00–6.00	5.04 (1.40), 3.00–7.00	<0.001
Child’s sex, *n* (%)					0.42
Female	147 (43)	125 (43)	107 (45)	83 (51)	
Male	192 (57)	164 (57)	133 (55)	81 (49)	
Premature, *n* (%)	37 (11)	30 (10)	20 (8.3)	12 (7.3)	0.51
Underweight, *n* (%)	39 (12)	33 (11)	25 (10)	19 (12)	0.98
Job, *n* (%)					0.99
Employed	172 (51)	149 (52)	120 (50)	84 (51)	
Unemployed or on leave of absence	167 (49)	140 (48)	120 (50)	80 (49)	
Home income, *n* (%)					>0.99
less than 2 million yen	7 (3.2)	7 (3.7)	7 (4.5)	5 (4.5)	
2–4 million yen	54 (25)	47 (25)	38 (25)	26 (24)	
4–6 million yen	67 (31)	51 (27)	45 (29)	36 (33)	
6–8 million yen	39 (18)	39 (21)	27 (18)	17 (15)	
8–10 million yen	35 (16)	31 (16)	26 (17)	18 (16)	
10–15 million yen	13 (5.9)	11 (5.8)	10 (6.5)	7 (6.4)	
More than 15 million yen	4 (1.8)	4 (2.1)	1 (0.6)	1 (0.9)	
Unknown	120	99	86	54	

*^1^One-way ANOVA; Pearson’s Chi-squared test.*

### Materials

#### Infant Crying Questionnaire

The Infant Crying Questionnaire (ICQ) was used to measure mothers’ beliefs or response intentions to infant crying. This questionnaire contains 31 items based on two secondary factors and four first-order factors. Although the original version of the ICQ assumed five first-order factors, including directive control in parent-oriented beliefs, subsequent studies including the original author’s work have used only four subscales by excluding the directive control factor (Leerkes et al., 2017; Pruitt et al., 2020). In the present study, we also excluded directive control. Infant-oriented beliefs include attachment and communication, while parent-oriented beliefs include minimization and spoiling for crying infants. Infant-oriented items include concerns about the infant’s welfare, a desire to help the infant, or empathy for the infant (e.g., “I felt sad for the baby”). Parent-oriented items include self-focused concerns, negative and avoidance reactions toward the infant, or responses that are of interest or importance to the mother but not the infant (e.g., “All that crying made me feel nervous”). Each item is rated on a 5-point scale ranging from 1 (never) to 5 (always). As there had not been a Japanese version of the ICQ, the original version of the ICQ was translated into Japanese by the first author after obtaining permission from the original authors, and then back-translated by an English proofreading company. Prior to this study, the reliability and validity of the Japanese version of the ICQ were confirmed through confirmatory factor analyses and correlation analyses with related questionnaires in different participants than in this main study. These results are reported in the Supplementary Material. Internal consistency reliability of each ICQ subscale in the current sample as assessed by Cronbach’s alpha across the four time points was confirmed as adequate (Attachment α = 0.78–0.88, Communication α = 0.66–0.75, Minimization α = 0.78–0.80, and Spoiling α = 0.84–0.87).

#### The Short Form of Japanese Version of Infant Behavior Questionnaire-Revised

The Japanese version of the Infant Behavior Questionnaire-Revised (IBQ-R) consists of 85 items that are responded to using a 7-point scale, which reflects the frequency within the last week of infant reactions to certain situations. The 85 items included 14 dimensions of infant behavior (activity level, distress to limitations, fear, duration of orienting, smiling and laughter, high-intensity pleasure, low-intensity pleasure, soothability, falling reactivity, cuddliness, perceptual sensitivity, sadness, approach, and vocal reactivity). Activity level, distress to limitations, perceptual sensitivity, high-intensity pleasure, vocal reactivity, and fear dimensions formed the “surgency” cluster; low-intensity pleasure, smiling and laughter, activity, duration of orienting, and cuddliness formed the “negative emotionality” cluster (all items are inverted); and sadness (inverted), falling reactivity, and soothability formed the “orienting” cluster. Each cluster forms a subscale. The reliability and validity of this Japanese version was confirmed in a previous study (Nakagawa et al., 2009). In the present study, internal consistency reliability of the three IBQ-R subscales across the four time points was confirmed as adequate (Surgency α = 0.88–0.92, Nevative emotionality α = 0.71–0.83, and Orienting α = 0.64–0.78).

### Statistical Analysis

We first sought to examine cross-sectional effects of infant age on maternal beliefs about infant crying across the four waves. A regression analysis was conducted for each wave entering ICQ scores as dependent variables and the infant monthly age as the independent variable. Next, the LGM was employed to examine the longitudinal trajectories of infant-oriented and parent-oriented beliefs about infant crying over the 4-month postpartum period. LGM is a multivariate statistical method within the framework of structural equation modeling that allows for the modeling of repeated measures data to estimate intra-individual growth patterns over time. In this analysis, the latent variables of intercept and slope are constructed to predict the repeatedly observed variables. If the slope estimate is significant, it means that there is an overall longitudinal change. If the variance of the slope is significant, it means that there is an individual difference in the process of change of the observed variables.

Given the above-mentioned conditions, the following additional analyses were conducted to examine the individual differences in the change process in more detail. First, the association between changes in maternal beliefs about infant crying and infant temperament was analyzed using parallel LGM, which integrated three LGM s for each outcome variable and analyzed the cross-sectional, prospective, and parallel associations among latent variables. In order to see if a change would be predicted by other variables, we focused mainly on prospective associations. Finally, we conducted the GMM. This is an analysis that assumes latent classes in the basic LGM. The Bayesian information criterion (BIC) is used to estimate the appropriate number of classes (Jung and Wickrama, 2008). Finally, we also examined whether infant temperament was involved in the class classification. The R 3.6.1 (R Core Team, 2019), lavaan package (Rosseel, 2012) and flexmix package (Gruen and Leisch, 2008) were used for the analyses. We regarded *p* < 0.05 as statistically significant for each analysis, using two-tailed testing.

## Results

Participants who chose the same option for all questions were excluded from the analyses; two participants were excluded from Wave 1, zero from Wave 2, one from Wave 3, and one from Wave 4.

### Cross-Sectional Results

To examine the effects of infant age on maternal beliefs about infant crying, regression analysis was conducted separately for each wave (Table 2). In Wave 1, infant-oriented beliefs increased and parent-oriented beliefs decreased with the infant’s increase in monthly age. In the other waves, there was no considerable association between infant crying beliefs and monthly infant age. Next, infant status (premature and underweight) were included as covariates in each model. Dummy variables were created for prematurity and underweight with 0 or 1. We found that the results were similar to the no-covariate models (Supplementary Table 3).

**TABLE 2:** Results of regression analysis predicting infant crying questionnaire (ICQ) from child age in each wave.

		Infant-oriented beliefs		Parent-oriented beliefs	
Group	Characteristic	Beta (95% CI)^1^	*p*-value	Beta (95% CI)^1^	*p*-value
Wave 1	Child age	0.04 (0.01 to 0.07)	0.010	–0.08 (–0.13 to –0.02)	0.006
Wave 2	Child age	0.03 (–0.01 to 0.06)	0.10	–0.02 (–0.08 to 0.04)	0.47
Wave 3	Child age	0.01 (–0.04 to 0.05)	0.80	0.04 (–0.03 to 0.11)	0.26
Wave 4	Child age	–0.03 (–0.08 to 0.03)	0.33	0.07 (–0.02 to 0.16)	0.12

*^1^CI, confidence interval.*

### Basic Latent Growth Analysis

We used latent growth analysis to test the hypothesis that maternal beliefs about infant crying would change within individuals. First, the scores at the four time points for beliefs about infant crying were fitted to a univariate LGM.

Regarding the change in infant-oriented beliefs, the LGM provided adequate fit to the observed data [χ^2^ (5) = 4.421, *p* = 0.490; comparative fit index (*CFI*) = 1.000; root mean square error of approximation (*RMSEA*) = 0.000]. The estimated mean level for the sample at Wave 1 was significantly different from zero [*M* = 4.554, 95% CI = (4.497, 4.612), *p* = 0.000], and there were significant individual differences [σ^2^ = 0.088, 95% CI = (0.055, 0.121), *p* = 1.68e-07]. The sample as a whole did not show significant developmental change [*M* = –0.004, 95% CI = (–0.0267, 0.018), *p* = 0.714], nor individual differences in growth rates were observed in infant-oriented beliefs [σ^2^ = 0.006, 95% CI = (–0.000436, 0.012), *p* = 0.069].

Regarding parent-oriented beliefs, the univariate LGM provided an adequate fit to the observed data [χ^2^ (5) = 5.333, *p* = 0.377; *CFI* = 0.999; *RMSEA* = 0.0203]. The estimated mean level for the sample at Wave 1 was significantly different from zero [*M* = 2.327, 95% CI = (2.228, 2.425), *p* = 0], and there were significant individual differences [σ^2^ = 0.293, 95% CI = (0.197, 0.389), *p* = 1.99e-09]. Overall, the participants showed significant increases in parent-oriented beliefs [*M* = 0.039, 95% CI = (0.007, 0.07), *p* = 0.017]. In addition, there were significant individual differences in growth rates [σ^2^ = 0.018, 95% CI = (0.003, 0.032), *p* = 0.015].

Next, infant age and infant status (premature and underweight) were included as covariates in each model. We found that the results were similar to the no-covariate model in the LGM for infant-oriented beliefs. As with the model without covariates, the model fit was good [χ^2^ (11) = 5.333, *p* = 0.602; *CFI* = 1.000; *RMSEA* = 0.000]. The intercept was significantly different from zero [*M* = 4.538, 95% CI = (4.435, 4.641), *p* = 0.000], and there were significant individual differences [σ^2^ = 0.086, 95% CI = (0.053, 0.118), *p* = 0.000]. The sample did not show significant developmental change in infant-oriented beliefs [*M* = 0.021, 95% CI = (–0.019, 0.062), *p* = 0.296], or individual differences in growth rates [σ^2^ = 0.005, 95% CI = (–0.001, 0.011), *p* = 0.094]. In this model, none of the variables (infant age, prematurity, or underweight) were significantly associated with the intercept and slope (*p*s > 0.112).

For the LGM of parent-oriented beliefs, the inclusion of covariates partially changed the results. Model fit was good, as in the model without covariates [χ^2^ (11) = 9.980, *p* = 0.532; *CFI* = 1.000; *RMSEA* = 0.000]. The intercept was significantly different from zero [*M* = 2.282, 95% CI = (2.105, 2.459), *p* = 0.000], and there were significant individual differences [σ^2^ = 0.287, 95% CI = (0.192, 0.381), *p* = 0.000]. The variance of the slope remained significant in the model with covariates [σ^2^ = 0.016, 95% CI = (0.002, 0.030), *p* = 0.028]. The sample did not show significant developmental change in this model [*M* = 0.001, 95% CI = (–0.056, 0.057), *p* = 0.978]. Among the covariates, only infant age predicted the slope, albeit with a significant trend [*b* = 0.021, 95%CI = (–0.013, 0.044), *p* = 0.063]. Therefore, exploratory LGM analyses were conducted separately for each infant age group to examine the differences in the process of change in parent-oriented beliefs in detail. In the model with only 0-month-old infants, the model fit was not adequate [*N* = 31, χ^2^ (5) = 16.572, *p* = 0.005; *CFI* = 0.875; *RMSEA* = 0.273]. In the models for 1- and 2-month-old infants, the model fits were good, but the slope value did not reach significance [1-month-old: *N* = 27, χ^2^ (5) = 4.944, *p* = 0.423; *CFI* = 1.000; *RMSEA* = 0.000, *M* = –0.015, 95% CI = (–0.095, 0.066), *p* = 0.720; 2-month-old: *N* = 34, χ^2^ (5) = 6.05, *p* = 0.301; *CFI* = 0.986; *RMSEA* = 0.079, *M* = 0.018, 95% CI = (–0.043, 0.079), *p* = 0.562]. However, in the models for 3- and 4-month-old infants, the model fits were good and the slope variable was significant in each model [3-month-old: *N* = 38, χ^2^ (5) = 2.642, *p* = 0.423; *CFI* = 1.000; *RMSEA* = 0.000, *M* = 0.097, 95%CI = (0.024, 0.171), *p* = 0.009; 4-month-old: *N* = 31, χ^2^ (5) = 2.468, *p* = 0.781; *CFI* = 1.000; *RMSEA* = 0.000, *M* = 0.069, 95%CI = (0.017, 0.122), *p* = 0.010]. These results suggested that the increase in parent-oriented beliefs did not occur immediately after postpartum but after the third month postpartum.

### Parallel Growth Modeling

Next, we conducted multivariate parallel LGM to test for associations between changes in infant- and parent-oriented beliefs and changes in infant temperament, including surgency, negative emotionality, and orienting in the postpartum period. Table 3 presents the correlations between latent growth factors in the models for (a) surgency, (b) negative emotionality, and (c) orienting. In the prospective association between temperament and mothers’ beliefs, higher surgency temperament was negatively associated with changes in infant-oriented beliefs and positively associated with changes in parent-oriented beliefs. This suggests that parents of children who showed high surgency as a temperament were more likely to experience a gradual decline in infant-oriented beliefs and an increase in parent-oriented beliefs about their infant’s crying. There was no effect of other temperaments on beliefs about crying. Additionally, there was no effect of crying beliefs on temperament development. Prospective relations between infant surgency and infant- and parent-oriented beliefs persisted after controlling for infant age, prematurity, and underweight (Supplementary Table 4).

**TABLE 3:** Parameter estimates from the parallel latent growth models.

		(a) Surgency			(b) Negative emotionality			(c) Orientation		
		Estimate (b)	95% CI		Estimate (b)	95% CI		Estimate (b)	95% CI	
Cross-sectional association	Intercept (IO) ∼ Intercept (Temperament)	0.023	–0.027	0.073	–0.061	–0.113	–0.008	–0.014	–0.042	0.014
	Intercept (PO) ∼ Intercept (Temperament)	0.059	–0.028	0.146	0.036	–0.054	0.126	0.066	0.016	0.116
Prospective association	Intercept (IO) ∼ Slope (Temperament)	0.008	–0.006	0.022	–0.005	–0.022	0.012	0.000	–0.012	0.011
	Intercept (PO) ∼ Slope (Temperament)	–0.014	–0.038	0.011	0.004	–0.026	0.034	–0.014	–0.034	0.0055
	Intercept (Temperament) ∼ Slope (IO)	–0.049	–0.070	–0.028	0.008	–0.012	0.029	–0.007	–0.019	0.004
	Intercept (Temperament) ∼ Slope (PO)	0.036	0.007	0.064	0.016	–0.013	0.045	0.004	–0.011	0.020
Parallel association	Slope (IO) ∼ Slope (Temperament)	0.009	0.0035	0.015	–0.004	–0.011	0.0029	0.000	–0.005	0.004
	Slope (PO) ∼ Slope (Temperament)	–0.002	–0.01	0.0056	0.001	–0.009	0.01	0.011	0.005	0.018
Chi-square		70.958	0.034^a^		58.911	0.209^a^		72.899	0.024^a^	
CFI		0.982			0.991			0.973		
RMSEA		0.050			0.032			0.052		

*CI, confidence interval; IO, infant-oriented beliefs; PO, parent-oriented beliefs, a = p value.*

### Growth Mixture Modeling

**FIGURE 1:**
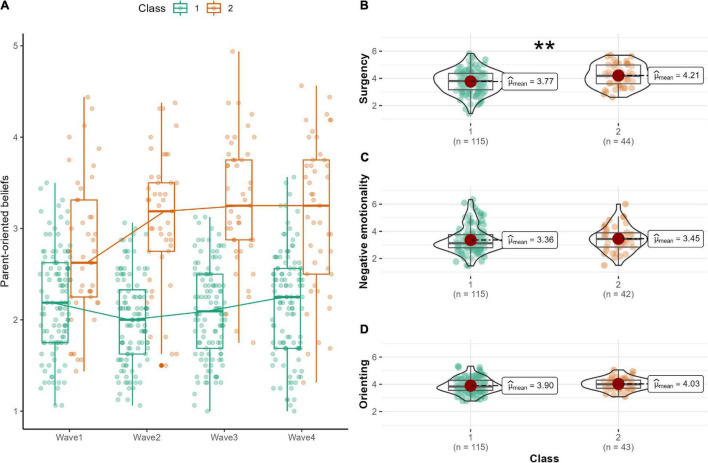
**(A)** Two-class growth mixture modeling for parent-oriented beliefs. **(B)** Difference in surgency scores between the two classes. **(C)** Difference in negative emotionality scores between the two classes. **(D)** Difference in orienting scores between the two classes. **p < 0.01.

The results of the basic LGM showed that there was significant individual differences in the change in parent-oriented beliefs. In the GMM, a latent class is set to divide the intercept and the slope into multiple clusters. The number of clusters was set from 1 to 5, and the BIC of each model was compared (Jung and Wickrama, 2008). As a result, the two-class model was found to have the lowest BIC and the best fit (Table 4). The scores for parent-oriented beliefs, divided into two classes, are shown in Figure 1A. Parent-oriented beliefs comprised two groups: one that showed an increase and one that did not show an increase. Finally, we conducted a one-way analysis of variance with surgency scores as the dependent variable and class as the independent variable to examine whether infants’ surgency temperament, which was shown to predict changes in parent-oriented beliefs in the parallel LGM, was also involved in group partitioning. The analysis revealed that infants in Class 2 (the group with increased parent-oriented beliefs) had significantly higher surgency temperament (*t* = –2.731, *p* = 0.00783, Figure 1B). The same analyses were conducted for the other aspects of temperament, but no differences were found between the groups (Negative emotionality: *t* = –1.214, *p* = 0.229; Orienting: *t* = –1.276, *p* = 0.205, Figures 1C,D). After controlling for covariates (infant age, prematurity, and underweight), the model with two classes had the lowest BIC (Supplementary Table 5). The results of ANOVA based on the results of the class classification after controlling for covariates showed only a significant difference in surgency between the classes as well as no-covariates analyses (Surgency: *t* = –2.95, *p* = 0.006; Negative emotionality: *t* = –0.284, *p* = 0.778; Orienting: *t* = –1.08, *p* = 0.290).

**TABLE 4:** Fit indices for growth mixture modeling analysis examining changes of parent-oriented beliefs (*N* = 161).

Number of classes	BIC	Sample size by class based on most likely membership				
1	1,178	161				
2	1,150	116	45			
3	1,195	88	68	5		
4	1,245	51	7	46	57	
5	1,295	29	44	7	76	5

*BIC, Bayesian information criterion.*

## Discussion

The present study adopted a longitudinal research design to examine whether maternal beliefs and response intentions about infant crying change within individuals. To the best of our knowledge, this is the first study to model longitudinal changes in maternal beliefs about infant crying and to show the relationship between individual differences in these changes and infant temperament. The results showed that maternal parent-oriented beliefs about infant crying increased intra-individually. However, when the infant age at Wave 1 was controlled, this change was no longer significant, and a significant increase was observed when the infant was 3 months or older at Wave 1. In addition, substantial individual differences were found in the growth processes of parent-oriented beliefs. Furthermore, in terms of the parallel relationship with infant temperament development, mothers of children with higher surgency, which reflects greater responsiveness and activity level to external stimuli, were more likely to have decreased infant-oriented beliefs and increased parent-oriented beliefs. There was no prospective association between other temperaments and maternal beliefs. Finally, it was shown that the change in parent-oriented beliefs was not uniform, but was divided into two groups, one that increased and one that did not, and that high infant surgency was involved in this clustering. The relationship between the level of infant surgency and the process of change in maternal parent-oriented beliefs did not change after controlling for infant age.

The cross-sectional regression analyses revealed a positive association between infant age and infant-oriented beliefs and a negative association between infant-oriented beliefs and parent-oriented beliefs only in Wave 1 (0–4 months). This result appears to be consistent with the results of a previous study, which found that immediately after birth, infants cry indiscriminately as a physiological reflex, but gradually crying becomes a means of social communication (Bell and Ainsworth, 1972). In the early stages, first-time mothers respond in a trial-and-error fashion regardless of the type of crying, but they gradually start identifying what each type of crying implies and take individualized action for each situation by 16 weeks (Drummond et al., 1993). In their cross-sectional research study, Parsons et al. (2017) showed that the functional brain activity in the amygdala and orbitofrontal cortex to infant crying correlated with infant’s monthly age. These areas are involved in empathy, reward processing, and the core network of reflective caregiving. It is possible that mothers increase their infant-oriented beliefs as they get better at understanding the reasons and the infant’s emotions behind each type of crying during the 0–4 month period.

In the longitudinal modeling, the present study showed a gradual increase in parent-oriented beliefs, which may reflect a gradual shift in focus from the infant’s crying to the self and withdrawing from always being preoccupied with the infant (Winnicott, 1956). However, the changes were not uniform, and mothers of infants aged 0–2 months at Wave 1 did not show an increase in parent-oriented beliefs across the four time points. This result was partially consistent with the results of the cross-sectional regression analyses above. Immediately after birth, the infant is vulnerable and crying is a signal function (Soltis, 2004), at which time it is considered adaptive for infant survival to empathize and respond immediately to crying. According to attachment theory, postpartum mothers are rewarded by the infant’s cries and are immediately motivated to approach and make physical contact with them (Bowlby, 1969). Winnicott (1956) described this phenomenon as an “almost illness” and argued for the necessity of gradual separation of mother and child. For 0–5 months postpartum, mothers might be immersed in the infant’s crying and may not yet shift their attention to themselves. However, for 3–7 months postpartum, the sensitivity to infant crying and the sense of obligation to make the infant stop crying may gradually decrease, and mothers might become more interested in themselves. Recent studies have reported that mothers may be able to partially judge the urgency of crying (Kurth et al., 2014) and may selectively ignore “crying out” more frequently with their experience (Bilgin and Wolke, 2020). The discrepancy between the results of the cross-sectional data analysis and the longitudinal data analysis can be attributed to the dynamic developmental changes that are difficult to be captured by the cross-sectional study design (Kraemer et al., 2000; Murayama, 2012). While McElwain and Booth-Laforce (2006) showed that maternal sensitive responses to infant distress predicted the formation of secure attachment, a recent study reported that there was no significant association between the frequency of leaving infants to cry it out and the formation of secure attachment. While interventions to increase sensitivity to infant distress are being developed to support mothers (e.g., Hepworth et al., 2020), it has also been suggested that mothers themselves are increasingly able to maintain a certain distance from infant crying to manage their own mental health (Kurth et al., 2014). Although the appropriate behavior for infant crying is still being debated, the desired response may depend on the mother-child relationship, and the age and temperament of the child. In research on parenting behavior, the danger has been pointed out that past studies that were not replicable or involved only small samples have been exaggeratedly cited and have become the theoretical basis for the development of recent interventions, especially those related to the treatment of infant crying (Van IJzendoorn and Bakermans-Kranenburg, 2021). In this study, we do not intend to discuss whether infant-oriented beliefs or parent-oriented beliefs are preferable. However, it seems important for mothers to distance themselves appropriately from crying as their infants grow and develop, and to show increased concern for themselves as well as for their infants (Winnicott, 1956; Kurth et al., 2014). In this regard, an increase in parent-oriented beliefs may be adaptive and lead to better management of the mother’s own mental health, especially when she has an active and difficult-to-care-for child. However, previous studies have shown that high parent-oriented beliefs were associated with externalizing and internalizing problem behaviors in children (Haltigan et al., 2012). In future research, it may be helpful to consider the interaction between parent- and infant-oriented beliefs, as well as the inverted U-shaped relationship between parent-oriented beliefs and mother-infant outcomes. It will be important for future intervention studies to take a longitudinal and statistical approach to elucidate the factors that determine the response to crying. It will be useful for both fundamental and clinical research to examine what the desired response to crying is for both mothers and children through a more long-term and comprehensive study design in the future.

The results of the univariate LGM showed substantial individual differences in growth patterns in parent-oriented beliefs. The results of the multivariate LGM and GMM revealed that the process of change in parent-oriented beliefs was not uniform, and that infant surgency contributed to these individual differences. Mothers of infants with higher surgency in Wave 1 were more likely to have a subsequent decrease in infant-oriented beliefs, while parent-oriented beliefs increased. Some participants were grouped into a cluster with increased parent-oriented beliefs; these participants rated their children as having a high surgency temperament at Wave1. The current results are consistent with a previous study that found that mothers engaged in caregiving behaviors less when infant temperament was high in surgency (Planalp et al., 2013). Surgency is a superordinate factor of the infant temperament, which is characterized by sensation seeking, high activity level, impulsivity, and positive emotionality. High surgency has been associated with more challenging care of infants (McBride et al., 2002), and mothers of infants with high activity tracked their infant’s location more closely (Kochanska and Aksan, 2004), which may reflect that the mothers needed to observe their child more and engage in proactive control. Infants high on surgency are more challenging to control, and children with high surgency are at higher risk for developmental problems such as externalizing problems, including aggression, conduct disorder, and attention-deficit/hyperactivity disorder (Putnam, 2012). Furthermore, the acoustic characteristics of crying and infants’ temperament have been examined in previous studies; for example, the cries of infants with high surgency were higher in the fundamental frequency than those infants who were low on surgency (Huffman et al., 1994), and an increase in fundamental frequency was associated with more harsh maternal responses (Out et al., 2010). Daily maternal experience of mothers who have infants that cry with high fundamental frequencies might decrease their caregiving and empathic response to crying, and avoidant feelings may increase. As mentioned above, while infants high on surgency are at a higher risk for externalizing problems, it has been found that children of caregivers with high parent-oriented beliefs were also more likely to manifest externalizing problems after 6 months (Haltigan et al., 2012). The current results suggest that a high level of surgency may lead to more parent-oriented beliefs and change parenting behaviors, which may lead to conduct problems in children in the long-term. Although the present study did not find an effect of maternal beliefs about crying on children’s change in temperament, there may be socio-psychological affects in the long term, and thus, bidirectional associations should be explored in the future.

The present study did not confirm the prospective associations between negative emotionality or orienting and change in beliefs about infant crying, which is in line with Planalp et al.’s (2013) findings that negative emotionality and orienting did not affect change in parenting behaviors in mothers, and that only surgency had an influence. Planalp et al. also found that infant surgency affected maternal playing behavior in addition to maternal caregiving, and approaching toys and a lot of laughter were also aspects of surgency. Although studies have shown that negative emotionality and orienting affected the way parents interact with their children or parenting style (Scaramella et al., 2008; Wittig and Rodriguez, 2019), it is possible that parenting behaviors closer to the temperament may be more susceptible to children’s temperament. In the Japanese version of the IBQ used in this study, the dimensions related to crying, such as distress to limitation and fear, were classified as surgency, and thus may have been most strongly associated with maternal beliefs about infant crying. While focusing on the response to crying is a strength of the present study, which is one of the most important parenting behaviors, other parenting behaviors and sensitivities might not necessarily show the same changes and relationships with temperament. It may be necessary in the future to apply the current methodology of longitudinal statistical modeling to other parenting behaviors.

One of the significant features of this study is that it was conducted on Japanese participants. In Japan, mothers and infants share a bed in many households (Shimizu et al., 2014), and parents are interdependent with their children, emphasizing infants’ sleep and a need for rapid gratification (Chen and Miyake, 1984). In contrast, in Western countries, parents value the growing independence of their children, giving them separate rooms and talking to them actively and frequently (Caudill and Weinstein, 1969; Chen and Miyake, 1984). In addition, there are cultural differences in children’s behavior between the East and West; American babies are more physically active and happily vocal, and more involved in the exploration of their bodies than Japanese babies (Caudill and Weinstein, 1969). In a study on infant temperament using the IBQ, it was shown that Japanese infants had lower levels of high intensity pleasure and vocal reactivity (but higher levels of fear), which are classified as surgency, than infants from other countries (Gartstein et al., 2010). Based on the present findings that higher surgency predicted an increase in parent-oriented beliefs, mothers of non-Japanese infants with higher levels of behavior may experience a steeper increase in parent-oriented beliefs than mothers in Japan. However, mother-infant communication through crying has been observed in mammals in general, and a common neurological mechanism for the parental response to crying has been found (e.g., Lonstein et al., 2015). In humans, the response to crying is to some extent culture-universal. A study of the maternal responses to infant crying conducted in 11 countries showed that affection, distraction, and nurturance responses to crying were not observed across cultures, whereas, talking to and picking up crying infants were observed in all cultures (Bornstein et al., 2017). Furthermore, using functional brain imaging, the authors have found that the supplementary motor area is commonly activated in mothers in response to crying across countries. Considering the above points, it is possible that the degree of change in beliefs about crying and the interrelationships with the infant temperament may differ between cultures, while the direction of change itself may be culturally universal. The process suggested by this study, of mothers becoming more concerned with themselves as infant development, has been noted in studies conducted in Western countries (e.g., Kurth et al., 2014; Bilgin and Wolke, 2020), which may reflect a culturally universal maternal adaptation process to infant crying. The present study is unique in that it was conducted in Japan, where research on parenting for crying is relatively small. In the future, it is necessary to discuss the culture-specificity and universality of the process of maternal change by conducting the longitudinal design as the present study in other countries.

### Limitation and Future Research

The present study adopted a robust longitudinal methodology and statistical modeling to examine the changes in maternal beliefs on infant crying. However, the following limitations exist and should be addressed in future research. First, the present study focused on subjective maternal beliefs about infant crying, and objective measures for mothers and infants were not obtained. However, the ICQ has been useful in prior research studies examining the sensitivities observed in mother-infant interactions, infant’s development in social behavior, individual differences in maternal insecure attachment, and depression that leads to individual differences in parenting (Leerkes et al., 2010, 2011; Haltigan et al., 2012; Pruitt et al., 2020). Additionally, the mother’s perception of crying—rather than observable factors of crying such as frequency of crying—may be more closely related to caregiver stress (Leerkes et al., 2015). Although the present study was not designed to examine parenting behavior itself or frequency of infant crying, future research should include observable objective factors of crying to comprehensively examine the relationship between the observed parenting behavior, frequency of crying, mother’s perception of infant crying, and developmental changes by using voice-activated digital recording devices or video-recording (Cabana et al., 2021). Second, in the present study, differences in mothers’ temperament were not assessed. Given 20–60% of temperament is inherited (Saudino, 2005), maternal individual variation in temperament may influence both changes in infant temperament and maternal beliefs about infant crying. Recently, a framework called a genetically informed design has been proposed (Taraban and Shaw, 2018), in which a common genetic background between mother and child determines both the infant’s temperament and parenting behavior. For example, Holmboe et al. (2011) found a dopamine receptor gene polymorphism (DRD4) was involved in the development of the infant’s temperament, while Leerkes et al. (2017) found it was also associated with both infant-oriented and parent-oriented beliefs. In other words, it should be noted that the similarity of genetic background and temperament between parents and their children may determine the bi-directionality of postpartum mother and infant development. Therefore, future studies should include both maternal and child genetic polymorphisms and temperament to get a comprehensive understanding of the directionality of the association. Finally, this study did not measure maternal psychopathology symptoms, which may have influenced both beliefs about crying and perceptions of the infant’s temperament. Since the early postpartum period has shown high prevalence rates of postpartum depression (O’Hara and McCabe, 2013; McCall-Hosenfeld et al., 2016), and the features of infant crying are associated with the occurrence and maintenance of depression (Radesky et al., 2013; Gabrieli et al., 2020), the interrelationships between postpartum depressive symptoms, beliefs about crying, and infant development should be examined in future studies.

## Conclusion

A small number of studies have sought to determine the change of maternal responses to infant crying within an individual in the first few months after delivery. This study used longitudinal data modeling to examine how beliefs about infant crying and behavioral intentions change in first-time mothers. The results showed that parent-oriented beliefs about infant crying increased gradually among mothers who had infants aged 3 months or older at Wave 1. In addition, the process of change was not uniform and was partly explained by surgency temperament. It has been suggested that how to respond to infant crying may depend on the development and temperament of the infant. While infant crying is an important factor in infant survival and development, it can also be stressful for mothers (Reijneveld et al., 2004; Soltis, 2004). Therefore, interventions for mothers are necessary, but there is a danger of basing these interventions on findings that have low reproducibility (Van IJzendoorn and Bakermans-Kranenburg, 2021). The present findings indicate that mothers of children with high levels of surgency show increased parent-oriented beliefs. If this is an adaptive response, as discussed above, it could lead to increased parenting stress in parents who are unable to increase parent-oriented beliefs despite their child showing a high level of surgency. Therefore, in such cases, mindfulness parenting training, which trains parents to reduce parental preoccupation with ruminative negative thinking and to increase self-nourishing attention (e.g., Duncan et al., 2009; Shorey and Ng, 2021), may be more effective for these parents than training to increase their sensitivity to their children’s distress. In the future, we must take into account the possibility that the type of support needed may change according to the characteristics of the mother and child. The methodology and results of this study using longitudinal modeling contribute to a fundamental understanding of mothers’ responses to infant crying and to future practice for specific individual interventions.

## Data Availability Statement

The data, codes and Rmarkdown files are publicly available on the FigShare (https://doi.org/10.6084/m9.figshare.14050583).

## Ethics Statement

The studies involving human participants were reviewed and approved by the Ethics Committees of Graduate School of Education, Kyoto University. The patients/participants provided their written informed consent to participate in this study.

## Author Contributions

DH and MK carried out the survey and analyzed the data. DH wrote the manuscript. DH, MN, and MK conceived the study, interpreted the results, reviewed, and proofed the manuscript.

## Conflict of Interest

The authors declare that the research was conducted in the absence of any commercial or financial relationships that could be construed as a potential conflict of interest.

## Publisher’s Note

All claims expressed in this article are solely those of the authors and do not necessarily represent those of their affiliated organizations, or those of the publisher, the editors and the reviewers. Any product that may be evaluated in this article, or claim that may be made by its manufacturer, is not guaranteed or endorsed by the publisher.
