# Supplementary material for: Longitudinal Study of Maternal Beliefs About Infant Crying During the Postpartum Period: Interplay With Infant’s Temperament
Source: Front Psychol. 2021 Dec 16;12:786391. doi: 10.3389/fpsyg.2021.786391 (PMC8716885; doi:10.3389/fpsyg.2021.786391)
Supplement: Supplementary file 1 [file Data_Sheet_1.docx]

Supplementary Material

# The development of Japanese version of Infant Crying Questionnaire

To develop the Japanese version of Infant Crying Questionnaire (ICQ), a questionnaire survey was administered to 235 mothers, who were independent of the participants of the main study. All participants were mothers, and the mean age was 31.702 (SD = 4.804). All of the participants’ youngest children were less than two years-old (mean monthly age = 13.498, SD = 7.364). Among them, the primiparous mothers were 133. All participants were recruited using Crowdworks (a crowdsourcing service in Japan).

The correlations between ICQ and empathy, aggression, and infant temperament were examined for construct validity. Trait empathy was assessed using the Interpersonal Reactivity Index,1 which measures four aspects of empathy: empathic concern (EC), personal distress (PD), perspective taking (PT), and fantasy (FS). In the original version, it was shown that the empathic feeling after watching a video clip of a crying baby positively correlated with Communication and negatively correlated with Spoiling and Minimization.2,3 Empathic concern, an aspect of empathy, is an other-oriented empathic emotion, similar to infant-oriented beliefs.1,2 On the other hand, personal distress is a self-oriented emotion that arises from observing the distress of others and is similar to parent-oriented beliefs. Therefore, it was hypothesized that empathic concern was positively associated with infant-oriented beliefs and personal distress was positively associated with parent-oriented beliefs, respectively.

The Buss-Perry Aggression Questionnaire4 was used to assess participants’ trait aggression. Responses were provided using a 5-point-Likert scale ranging from 1 (extremely uncharacteristic of me) to 5 (extremely characteristic of me). The BPAQ has four subscales (Physical aggression(PA), Verbal aggression (VA), Angry and Hostility). We hypothesized that Angry in BPAQ and Minimization would be positively correlated because anger rating into video clip of crying baby positively correlated with Minimization,2 and parent-child aggression risk and parent-oriented beliefs has also been positively correlated.5

The infants’ temperament was measured using the IBQ-R as well as the main study. In the original version of the ICQ, parent-oriented beliefs have been shown to be positively related to infants’ internalizing and externalizing problem behaviors after six month.2 Because it has been pointed out that negative emotionality promotes internalizing and externalizing behaviors and orientation inhibits them,6,7 we hypothesized that there would be a positive association between parent-oriented beliefs and negative emotionality, and a negative association between Orientation and negative emotionality in this study.

A confirmatory factor analysis assuming four first-order factors and two second-order factors showed an acceptable goodness of fit (CFI = 0.886, RMSEA = 0.054, see S. Table 1). However, two items had very low factor loadings (< 0.03), so we excluded them and performed another confirmatory factor analysis. After they were removed, the goodness of fit and omega coefficient improved (CFI = 0.911, RMSEA = 0.052). Therefore, it is recommended to use differently depending on the purpose of the study; for example, it can be used without the two items when a more reliable measure of parental beliefs towards infant crying in needed, or used with them when an international comparison of parental beliefs is needed. The former was used in the following analysis and in the main analysis of this study.

The mean and standard deviation of each ICQ score and the correlation coefficients with each questionnaire were shown in S. Table 2. Regarding the correlation analysis between the factors of ICQ, the factors in the infant-oriented beliefs and the factors in the parent-oriented beliefs were generally negatively correlated, similar to the results of original study.2

With regard to the relationship between the IRI and the ICQ, there were positive correlations between the empathic concern score and Attachment and Communication scores, as was hypothesized. At the same time, personal distress scores, which are self-oriented attitudes, were positively correlated with parent-oriented beliefs to infant crying. With regard to the aggression questionnaire, as also hypothesized, there were positive correlations between parent-oriented beliefs and physical aggression and angry scores. Negative emotionality was positively correlated with Parent-oriented beliefs, while Orientation showed an inverse correlation, consistent with the hypothesis. From the above results, it was concluded that the Japanese version of ICQ can measure similar constructs to the original version.

| Supplementary Table 1. The content of each item in the Japanese version of ICQ and the results of the confirmatory factor analysis | | | | | |
| --- | --- | --- | --- | --- | --- |
|  | ICQ.item | Factor loading | | Omega | |
| Infant-oriented beliefs | |  |  |  |  |
|  | Attachment | 0.91 | 0.91 | 0.81 | 0.81 |
| 1 | 赤ちゃんを安心させてあげたいと思う | 0.57 | 0.57 |  |  |
| 2 | 赤ちゃんをより気分よくさせてあげたいと思う | 0.56 | 0.56 |  |  |
| 3 | 赤ちゃんを慰めてあげたいと思う | 0.56 | 0.56 |  |  |
| 4 | 赤ちゃんは守られていて大切にされているのだと感じさせてあげたいと思う | 0.60 | 0.60 |  |  |
| 5 | 私が頼れる存在だと赤ちゃんに感じさせてあげるだろう | 0.60 | 0.60 |  |  |
| 6 | 助けを求められる存在として私がいることを赤ちゃんに知ってほしいと思う | 0.54 | 0.54 |  |  |
| 7 | 私が赤ちゃんの感じていることを気にかけているということを，赤ちゃんに感じさせてあげるだろう | 0.67 | 0.67 |  |  |
| 8 | 赤ちゃんに安心や安全を感じさせようとするだろう | 0.61 | 0.61 |  |  |
|  | Communication | 0.76 | 0.76 | 0.59 | 0.59 |
| 9 | 赤ちゃんは私に何かを教えようとしていると思う | 0.56 | 0.56 |  |  |
| 10 | 赤ちゃんは私に何かを伝えようとしていると思う | 0.66 | 0.66 |  |  |
| 11 | 赤ちゃんが泣いているのには何かしらの理由があると思う | 0.49 | 0.49 |  |  |
| Parent-oriented beliefs | |  |  | 0.78 | 0.81 |
|  | Minimization | 0.62 | 0.61 |  |  |
| 12 | 他に何もできなくなるので，泣き止んでほしいと思う | 0.65 | 0.65 |  |  |
| 13 | 他の人が嫌な思いをしないよう、泣き止ませたいと思う | 0.44 | 0.44 |  |  |
| 14 | 泣いてもどうにもならないので，泣き止んでほしいと思う | 0.81 | 0.81 |  |  |
| 15 | 泣き声は不快なので，すぐに泣き止ませたいと思う | 0.69 | 0.69 |  |  |
| 16 | どう対応すれば正しいのかわからないので，泣き止んでほしいと思う | 0.75 | 0.75 |  |  |
| 17 | 赤ちゃんは私をコントロールまたは操作しようとしているように思う | 0.41 | 0.41 |  |  |
| 18 | 赤ちゃんはただ関心を集めたいだけだと思う | 0.48 | 0.48 |  |  |
| 19 | 泣く正当な理由はないことを赤ちゃんに教えるだろう | 0.24 | 0.25 |  |  |
| 20 | 感情が昂っても大丈夫だと赤ちゃんに教えるだろう | -0.02 |  |  |  |
|  | Spoiling | 0.95 | 0.96 | 0.59 | 0.77 |
| 21 | 甘えすぎないように，赤ちゃんを泣いたままにする | 0.75 | 0.75 |  |  |
| 22 | 私に依存させすぎないように，赤ちゃんを泣いたままにする | 0.83 | 0.83 |  |  |
| 23 | 私は赤ちゃんを甘やかしてだめにしてしまうかもしれない | -0.02 |  |  |  |
| The factor loadings and omega coefficients represented the values which on the left when all items were used and on the right when two items were removed. | | | | | |

| Supplementary Table 2. Means, standard deviations, and correlations of the scales | | | | | | | | | | |
| --- | --- | --- | --- | --- | --- | --- | --- | --- | --- | --- |
|  | | Scale | Mean | SD | 1 | 2 | 3 | 4 | 5 | 6 |
| 1 | ICQ | Attachment | 4.5 | 0.46 | 1.00 | 0.48*** | -0.22*** | -0.30*** | 0.96*** | -0.27*** |
| 2 |  | Communication | 4.5 | 0.49 | **0.48***** | 1.00 | -0.11 | -0.25*** | 0.71*** | -0.16* |
| 3 |  | Minimization | 2.9 | 0.72 | **-0.22***** | **-0.11** | 1.00 | 0.48*** | -0.21** | 0.97*** |
| 4 |  | Spoiling | 2.3 | 1.01 | **-0.30***** | **-0.25***** | **0.48***** | 1.00 | -0.32*** | 0.69*** |
| 5 |  | IO | 4.5 | 0.42 | 0.96*** | 0.71*** | -0.21** | -0.32*** | 1.00 | -0.27*** |
| 6 |  | PO | 2.7 | 0.70 | -0.27*** | -0.16* | 0.97*** | 0.69*** | -0.27*** | 1.00 |
| 7 | IRI | EC | 3.6 | 0.57 | **0.31***** | **0.29***** | -0.04 | -0.12 | **0.34***** | -0.07 |
| 8 |  | PD | 3.3 | 0.71 | -0.01 | 0.07 | **0.39***** | **0.13*** | 0.01 | **0.36***** |
| 9 |  | PT | 3.1 | 0.62 | 0.26*** | 0.11 | -0.15* | -0.10 | 0.25*** | -0.16* |
| 10 |  | FS | 3.4 | 0.82 | 0.18** | 0.23*** | 0.12 | -0.02 | 0.22*** | 0.09 |
| 11 | BPAQ | PA | 2.5 | 0.76 | -0.24*** | -0.16* | **0.24***** | **0.22***** | -0.25*** | **0.26***** |
| 12 |  | VA | 3.0 | 0.66 | 0.07 | 0.09 | -0.01 | 0.00 | 0.08 | -0.01 |
| 13 |  | Anger | 3.0 | 0.89 | -0.05 | -0.05 | **0.22***** | **0.17**** | -0.06 | **0.23***** |
| 14 |  | Hostility | 3.0 | 0.74 | -0.10 | -0.05 | 0.25*** | 0.17** | -0.10 | 0.25*** |
| 15 | IBQ | Surgency | 5.8 | 1.08 | 0.04 | 0.14* | 0.17** | -0.02 | 0.07 | 0.14* |
| 16 |  | Negative emotionality | 2.8 | 0.77 | -0.11 | -0.16* | **0.19**** | **0.12** | -0.14* | **0.19**** |
| 17 |  | Orientation | 4.5 | 0.88 | 0.12 | 0.17** | **-0.40***** | **-0.16*** | 0.15* | **-0.37***** |
| IO = Infant-oriented beliefs, PO = Parent-oriented beliefs, EC = Empathic concern, PD = Personal distress, PT = Perspective taking, FS = Fantasy, PA = Physical aggression, VA = Verbal aggression | | | | | | | | | | |
| *** p < . 001, ** p < .01, * p < . 05 | | | | | | | | | | |
| Bold figures indicate the hypothesized correlations with respect to construct validity examinations. | | | | | | | | | | |

# Cross-sectional results

| Supplementary Table 3. Results of regression analysis predicting ICQ from child age in each Wave including covariates (prematurity and underweight) | | | | | |
| --- | --- | --- | --- | --- | --- |
|  | | Infant-oriented beliefs | | Parent-oriented beliefs | |
| Group | Characteristic | Beta (95% CI)^1^ | p-value | Beta (95% CI)^1^ | p-value |
| Wave1 | Child age | 0.04 (0.01 to 0.08) | 0.009 | -0.07 (-0.13 to -0.02) | 0.008 |
|  | Premature | -0.05 (-0.21 to 0.10) | 0.49 | 0.32 (0.07 to 0.58) | 0.014 |
|  | Underweight | -0.09 (-0.24 to 0.06) | 0.24 | -0.11 (-0.36 to 0.14) | 0.39 |
| Wave2 | Child age | 0.03 (0.00 to 0.07) | 0.085 | -0.02 (-0.09 to 0.04) | 0.44 |
|  | Premature | -0.08 (-0.25 to 0.09) | 0.36 | 0.23 (-0.06 to 0.53) | 0.12 |
|  | Underweight | -0.06 (-0.22 to 0.10) | 0.47 | 0.06 (-0.22 to 0.35) | 0.65 |
| Wave3 | Child age | 0.01 (-0.04 to 0.05) | 0.71 | 0.04 (-0.03 to 0.11) | 0.25 |
|  | Premature | -0.23 (-0.47 to 0.02) | 0.068 | 0.15 (-0.21 to 0.51) | 0.41 |
|  | Underweight | -0.04 (-0.26 to 0.18) | 0.72 | -0.11 (-0.43 to 0.22) | 0.51 |
| Wave4 | Child age | -0.03 (-0.08 to 0.03) | 0.33 | 0.07 (-0.02 to 0.16) | 0.11 |
|  | Premature | -0.11 (-0.42 to 0.19) | 0.47 | 0.20 (-0.30 to 0.71) | 0.43 |
|  | Underweight | 0.06 (-0.19 to 0.30) | 0.66 | -0.17 (-0.58 to 0.23) | 0.40 |
| ^1^CI = Confidence Interval | | | | | |

# Parallel growth modeling including covariates

| Supplementary Table 4. Parameter estimates from the parallel latent growth models including covariates (infant age, prematurity and underweight) | | | | | | | | | | |
| --- | --- | --- | --- | --- | --- | --- | --- | --- | --- | --- |
|  |  | (a) Surgency | | | (b) Negative emotionality | | | (c) Orientation | | |
|  |  | Estimate (b) | 95% CI | | Estimate (b) | 95% CI | | Estimate (b) | 95% CI | |
| Cross-sectional association | Intercept (IO) ~ Intercept (Temperament) | 0.017 | -0.030 | 0.065 | -0.051 | -0.097 | -0.004 | -0.013 | -0.040 | 0.015 |
|  | Intercept (PO) ~ Intercept (Temperament) | 0.054 | -0.028 | 0.136 | 0.047 | -0.032 | 0.127 | 0.068 | 0.019 | 0.117 |
| Prospective association | Intercept (IO) ~ Slope (Temperament) | 0.006 | -0.007 | 0.020 | -0.007 | -0.022 | 0.009 | -0.001 | -0.012 | 0.010 |
|  | Intercept (PO) ~ Slope (Temperament) | -0.012 | -0.036 | 0.012 | -0.002 | -0.029 | 0.024 | -0.014 | -0.034 | 0.005 |
|  | Intercept (Temperament) ~ Slope (IO) | -0.045 | -0.065 | -0.025 | 0.002 | -0.016 | 0.020 | -0.008 | -0.019 | 0.002 |
|  | Intercept (Temperament) ~ Slope (PO) | 0.030 | 0.003 | 0.057 | 0.025 | -0.001 | 0.051 | 0.006 | -0.010 | 0.021 |
| Parallel association | Slope (IO) ~ Slope (Temperament) | 0.010 | 0.004 | 0.015 | -0.002 | -0.008 | 0.004 | 0.000 | -0.004 | 0.005 |
|  | Slope (PO) ~ Slope (Temperament) | -0.003 | -0.011 | 0.004 | -0.002 | -0.010 | 0.007 | 0.011 | 0.004 | 0.017 |
| Chi-square |  | 95.645 | 0.019^a^ |  | 80.660 | 0.159^a^ |  | 96.430 | 0.024^a^ |  |
| CFI |  | 0.977 |  |  | 0.988 |  |  | 0.967 |  |  |
| RMSEA |  | 0.049 |  |  | 0.033 |  |  | 0.050 |  |  |
| CI = Confidence Interval, IO = Infant-oriented beliefs, PO = Parent-oriented beliefs, a = *p*-value | | | | | | | | | | |

# Growth mixture modeling including covariates

| Supplementary Table 5. it indices for growth mixture modeling analysis examining changes of parent-oriented beliefs including covariates (infant age, prematurity and underweight) (N = 161) | | | | | | |
| --- | --- | --- | --- | --- | --- | --- |
| Number of classes | BIC | Sample size by class based on most likely membership | | | | |
| 1 | 1194 | 161 |  |  |  |  |
| 2 | 1187 | 138 | 23 |  |  |  |
| 3 | 1229 | 117 | 25 | 19 |  |  |
| 4 | 1268 | 95 | 29 | 27 | 10 |  |
| 5 | 1334 | 61 | 56 | 26 | 10 | 8 |
| BIC = Bayesian information criterion | | | | | | |
